# miR-27b-3p Attenuates Muscle Atrophy by Targeting Cbl-b in Skeletal Muscles

**DOI:** 10.3390/biom12020191

**Published:** 2022-01-23

**Authors:** Xin Yang, Zhenhui Li, Zhijun Wang, Jiaao Yu, Manting Ma, Qinghua Nie

**Affiliations:** 1 State Key Laboratory for Conservation and Utilization of Subtropical Agro-Bioresources & Lingnan Guangdong Laboratory of Agriculture, College of Animal Science, South China Agricultural University, Guangzhou 510642, China; yangxin@stu.scau.edu.cn (X.Y.); lizhenhui@scau.edu.cn (Z.L.); zhijunwang@stu.scau.edu.cn (Z.W.); yujiaao@stu.scau.edu.cn (J.Y.); mamanting@stu.scau.edu.cn (M.M.); 2 Guangdong Provincial Key Lab of Agro-Animal Genomics and Molecular Breeding, and Key Laboratory of Chicken Genetics, Breeding and Reproduction, Ministry of Agriculture, Guangzhou 510642, China; 3 National-Local Joint Engineering Research Center for Livestock Breeding, Guangzhou 510642, China

**Keywords:** microRNA, Cbl-b gene, mice, muscle atrophy

## Abstract

As it is well known, muscle atrophy is a process in which protein degradation increases and protein synthesis decreases. This process is regulated by a variety of links. Among them, microRNAs play an essential role in this process, which has attracted widespread attention. In this paper, we find that miR-27b-3p and Cbl-b genes are significantly differentially expressed in the induced atrophy model. The dual-luciferase experiment and Western blot analysis confirmed that miR-27b-3p could regulate the expression of Cbl-b. In C2C12-differentiated myotubes, the overexpression of the Cbl-b gene showed that Cbl-b could upregulate the expression of MuRF-1 and Atrogin-1, which are related marker genes of muscle atrophy, at both the mRNA and protein levels, indicating that the Cbl-b gene can specifically affect muscle atrophy. The knockdown of the Cbl-b gene after C2C12-differentiated myotubes induced atrophy treatment can downregulate the expression of muscle-atrophy-related genes, indicating that manual intervention to downregulate the expression of Cbl-b has a certain alleviating effect on muscle atrophy. These data suggest that miR-27b-3p can regulate the expression of the Cbl-b gene and then exert a particular influence on muscle atrophy through the Cbl-b gene.

## 1. Introduction

Skeletal muscles are the most important part of the animal’s movement or keeping still, and their health status is closely related to the various behaviors of the animal. Muscle atrophy results from both protein degradation and decreased synthesis, caused by chronic diseases (such as diabetes), disuse (denervation, paralysis), aging, and cancer [1,2,3,4,5]. The area of muscle fibers decreases. At the same time, the phenotype of skeletal muscles changes significantly with the decrease in protein synthesis. The phenomenon is a complicated and height adjustment process [6,7,8]. This loss of muscle function may lead to a decline in quality of life, an increase in morbidity, and even death. At present, exercise is a more common way to resist muscle atrophy [7] and treating muscle atrophy with specific drugs is still an unresolved challenge. The process of muscle atrophy is regulated by many signaling pathways (such as the PI3K/AKT signaling pathway) and various regulatory factors (including DNA, non-coding RNA (ncRNA), and peptides). Among them, regulating gene expression with miRNAs as targets is also an essential part of this process.

MicroRNAs (miRNAs, miRs) are a type of endogenous non-coding RNA with regulatory functions, with a size of about 18–25 nucleotides. miRNA’s regulation of target genes is manifested at the post-transcriptional level, which can negatively regulate gene expression through the miRNA–mRNA interaction [9]. miRNAs are widely involved in cell proliferation, differentiation, apoptosis, and cell-cycle regulation and other processes. In recent years, studies have found that miRNAs are involved in the regulation of muscle atrophy. For example, miR-29b can cause various types of muscle atrophy by targeting IGF-1 and PI3K (P85a) [10]; miR-29c can improve the quality of skeletal muscle by inhibiting the expression of atrophy-related genes during the proliferation and differentiation of muscle cells and function [11]. To date, the research on miR-27b-3p has mainly focused on cancer drugs. For example, miR-27b-3p can regulate the drug resistance of colorectal cancer through the C-Myc/miR-27b-3p/ATG10 axis [12]. However, there are few reports about miR-27b-3p on muscle occurrence and damage repair.

Cbl-b is an E3 ubiquitin-protein ligase, which is involved in the regulation of a variety of cellular pathways. According to previous research reports, Cbl-b can play an important role in T-cell activation and control peripheral T-cell tolerance through multiple mechanisms during the immune process [13,14,15]. In addition, Cbl-b plays an important role in muscle atrophy [16,17,18,19]. In previous research reports, results suggested that Cbl-b was identified as an upregulated ubiquitin ligase in an atrophic mouse model. Cbl-b ubiquitinates and induces the degradation of IRS-1, a key intermediate of the IGF-1 signal transduction. IRS-1 is a key adapter protein in insulin-like growth factor-1 (IGF-1) signal transduction. In short, Cbl-b downregulates the IGF-1 signal in skeletal muscle under atrophic conditions [19,20,21].

In this study, we determine that miR-27b-3p and Cbl-b were significantly differentially expressed in the atrophy model through miRNA sequencing and mRNA sequencing. We also confirm through dual luciferase and Western blot experiments that Cbl-b is the target gene of miR-27b-3p. Finally, we overexpress the Cbl-b gene and knock down its expression in the atrophy model, proving that the Cbl-b gene can promote muscle atrophy. All in all, these data indicate that miR-27b-3p can regulate Cbl-b and thus affect muscle atrophy.

## 2. Materials and Methods

### 2.1. The Ethics Statement

The Institutional Animal Protection and Utilization Committee of South China Agricultural University approved the animal experiments conducted in this study. The experiment was carried out in accordance with established regulations and guidelines.

### 2.2. Cell Culture and Treatment

For the cell culture and transfection, the experiment used mouse skeletal muscle myoblasts (C2C12) as the research object, and the detection of mycoplasma contamination before use was negative. C2C12 cells were cultured in Dulbecco’s modified Eagle’s medium (DMEM) (Thermo Fisher Scientific, Waltham, MA, USA) containing 100 U/mL penicillin, 100 mg/mL streptomycin, 10% fetal calf serum and 4.5 gL^−1^ glucose. The cell incubator temperature was 37 °C, and the CO_2_ concentration was 5%.

The cells were seeded on a culture plate coated with 0.1% gelatin to induce differentiation. When the cell reached 70–80% confluence, the medium was replaced with a DMEM medium containing 2% horse serum (Thermo Fisher Scientific, MA, USA) to induce differentiation. After 4 days, a multinucleated myotube was formed. In order to induce muscle atrophy in vitro, dexamethasone was added to a 2% horse serum medium to a final concentration of 50 µM and incubated in a cell culture incubator for 24 h. After incubation, cells were taken to observe their morphology.

### 2.3. miRNA and mRNA Screen

We considered dexamethasone-treated cells as the experimental group, extracted RNA separately from the negative control group, and performed transcriptome sequencing.

### 2.4. Cell Transfection

Myotube transfection was performed with Liposome 3000 reagent (Thermo Fisher Scientific, MA, USA), according to the manufacturer’s instructions. The mimic and inhibitor of miR-27b-3p were synthesized by RiboBio (Guangzhou, China). The full-length coding sequence of the miRNA mouse Cbl-b overexpression plasmid was constructed by GeneCreate (Wuhan, China). The pcDNA3.1 empty vector was used as a control for the Cbl-b overexpression vector. Cbl-b siRNA was designed and synthesized by RiboBio. The transfection dose of mimic, inhibitor, Cbl-b gene overexpression plasmid and siRNA were all 150 nM. After myotubes were formed, they were transfected with miR-27b-3p mimics and inhibitors and Cbl-b gene overexpression plasmids, and total RNA was collected 48 h later. After the formation of myotubes, the transfected siRNA of the Cbl-b gene and dexamethasone were added simultaneously. The total RNA was collected after culturing for 36 h.

The sequences of the mimic and inhibitor of miR-27b-3p, and all siRNA sequences of Cbl-b are listed in Table S1.

### 2.5. Dual-Luciferase Reporter Assay

We synthesized the wild-type and mutated 3′UTR of Cbl-b in Tsingke Biotechnology (Beijing, China) and inserted them into the pmirGLO vector. The inserted 3′UTR sequence is in Table S3. Four combinations of wild-type vector (Cbl-b-WT)+ miRNA-27b-3p mimic, mutated vector (Cbl-b-MT)+ miRNA-27b-3p mimic, pmirGLO empty vector + NC mimic, and wild-type vector (Cbl-b-WT)+ NC mimic were co-transfected into the cell culture in 96-well plates. After 36h later, Dual-luciferase Reporter Assay System (Promega, Madison, WI, USA) was used to detect firefly and Renilla luciferase activity.

### 2.6. Experimental Animals

8-week-old male Kunming mice (n = 12) were purchased in Hunan SJA Laboratory Animal Co., Ltd. (Changsha, China) for in vivo experiments. After the three-day adaptation period, the mice were randomly divided into 2 groups. For the establishment of a model of muscle atrophy caused by dexamethasone, we set every 6 mice as a group, divided into dexamethasone treatment group (Dex) and control group (Con). In the Dex group, dexamethasone was added to the fresh water fed to the final concentration of 10 mg/kg per mouse per day for continuous treatment for 7 days. All mice were reared in an environment with a light-dark cycle of 12 h and an ambient temperature of 21–23 °C.

### 2.7. RNA Extraction, cDNA Synthesis, and Quantitative Real-Time PCR (qRT-PCR)

We used RNA iso (TaKaRa, Otsu, Japan) to extract total RNA from muscles and cells, according to the manufacturer’s instructions. Reverse transcription to synthesize RNA cDNA was performed using HiScript Q-RT SuperMix for qPCR (+gDNA wiper) (Vazyme, Nanjing, China). On the Bio-Rad CFX96 real-time detection system, the ChamQ Universal SYBR qPCR Master Mix (Vazyme) was used to perform qRT-PCR to detect the expression level of mRNAs. The data analysis of the results used the 2^−ΔΔCt^ Δ method to calculate the relative expression of genes. The reference genes in the experiment were GAPDH. The primers for real-time qPCR were designed by Premier Primer 5.0 software (Premier Biosoft, Palo Alto, CA, USA). Primers used for qPCR are listed in Table S2.

### 2.8. Western Blotting Assay

Cells or tissues were lysed with RIPA buffer (Beyotime, Hangzhou, China) containing a mixture of protease inhibitors. The total protein was quantified using BCA protein analysis kit (Beyotime). SDS-polyacrylamide gel electrophoresis (Beyotime) was used to separate the protein cleavage products. The main monovalent antibodies used were as follows: Cbl-b (1:500, Proteintech, Chicago, IL, USA), Atrogin-1 (1:1000, Bioss Antibodies, Beijing, China), MuRE-1 (1:1000, Proteintech). Protein was detected with BeyoECL Star reagent (Beyotime).

### 2.9. Hematoxylin and Eosin Staining (HE)

The mouse gastrocnemius and tibial anterior muscle tissues were taken, fixed with 4% paraformaldehyde, and embedded in paraffin. Tissue sections were stained with hematoxylin–eosin (HE), and then image analyzed.

### 2.10. Statistical Analysis

Based on at least three independent experiments, all experimental results are expressed as mean ± SEM. The statistical significance of the difference between the means was evaluated by performing an unpaired Student’s *t*-test, with statistical significance at *p* < 0.05; * *p* < 0.05; ** *p* < 0.01; *** *p* < 0.001.

## 3. Results

### 3.1. Screening of Differentially Expressed miRNAs in Atrophic Myotubes

**Figure 1:**
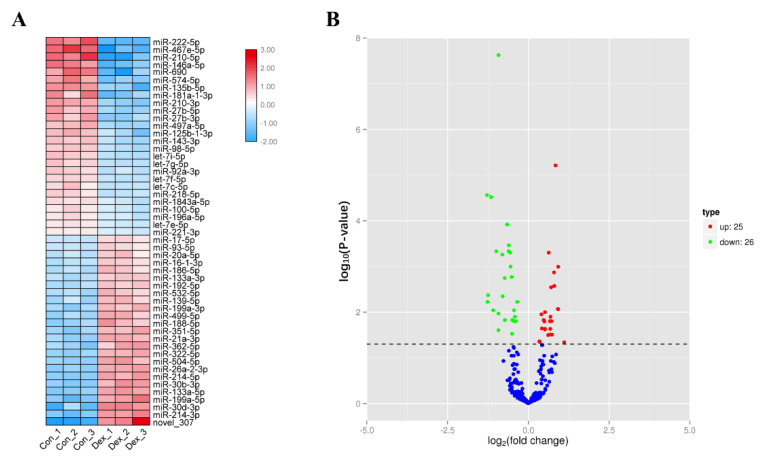
miRNA-seq analysis for the Dex group and Con group. (**A**) miRNA array shows miRNA dysregulation from Dex-induced models. (**B**) Volcano plot of DEMs from Dex VS Con.

To study differentially expressed miRNAs (DEMs) that have potential roles in muscle atrophy, we used 100 µM dexamethasone (Dex) to induce myotubes formed by C2C12 cell differentiation, causing them to produce atrophy (muscle atrophy). We extracted total cellular RNA and performed miRNA sequencing. Through sequencing, a total of 51 differentially expressed miRNAs were obtained between the Dex group and the Con group (Figure 1A), of which 26 miRNAs were downregulated, and 25 miRNAs were upregulated (Figure 1B); 50 were known miRNAs, and 1 was a newly discovered miRNA.

### 3.2. Transcriptome Sequencing Analysis to Identify and Screen Differentially Expressed Genes in Atrophic Myotubes

**Figure 2:**
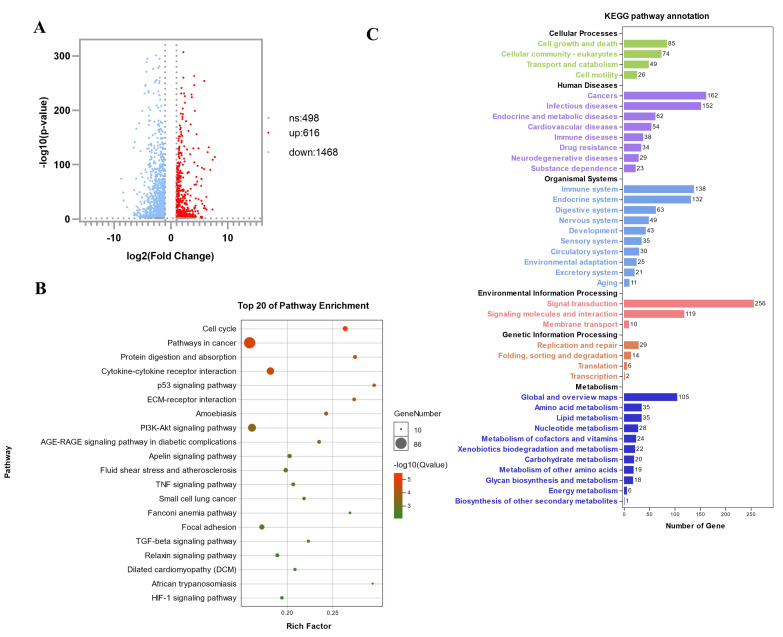
mRNA-seq analysis for the Dex group and Con group. (**A**) Volcano plot of DEGs from Dex VS Con. (**B**) GO enrichment analysis of DEGs. (**C**) Classification analysis of the KEGG pathway of DEGs.

In order to explore the differentially expressed genes (DEGs) of muscle atrophy, we performed transcriptome sequencing. In the myotubes formed by C2C12 differentiation, three dexamethasone-treated samples (Dex) and three blank-treated controls (Con) were collected for transcriptome sequencing. Through sequencing and later data analysis, we detected a total of 2582 genes, of which 2084 were differential expressed genes. Compared with the Con group, the Dex group had 1468 genes downregulated and 616 genes upregulated (Figure 2A). We performed a KEGG biological pathway classification and GO enrichment analysis for these differentially expressed genes. A total of 35 genes were classified as amino acid metabolism pathways, and 49 genes were classified as cellular transport and catabolic pathways (Figure 2B). Differentially expressed genes are enriched in many important metabolic pathways, including protein digestion and absorption, cytokine-cytokine receptor interaction, p53 signaling pathway, PI3K-Akt signaling pathway, and other pathways closely related to muscle atrophy (Figure 2C).

### 3.3. Joint Analysis and Interactive Network of Differentially Expressed miRNAs and Genes

**Figure 3:**
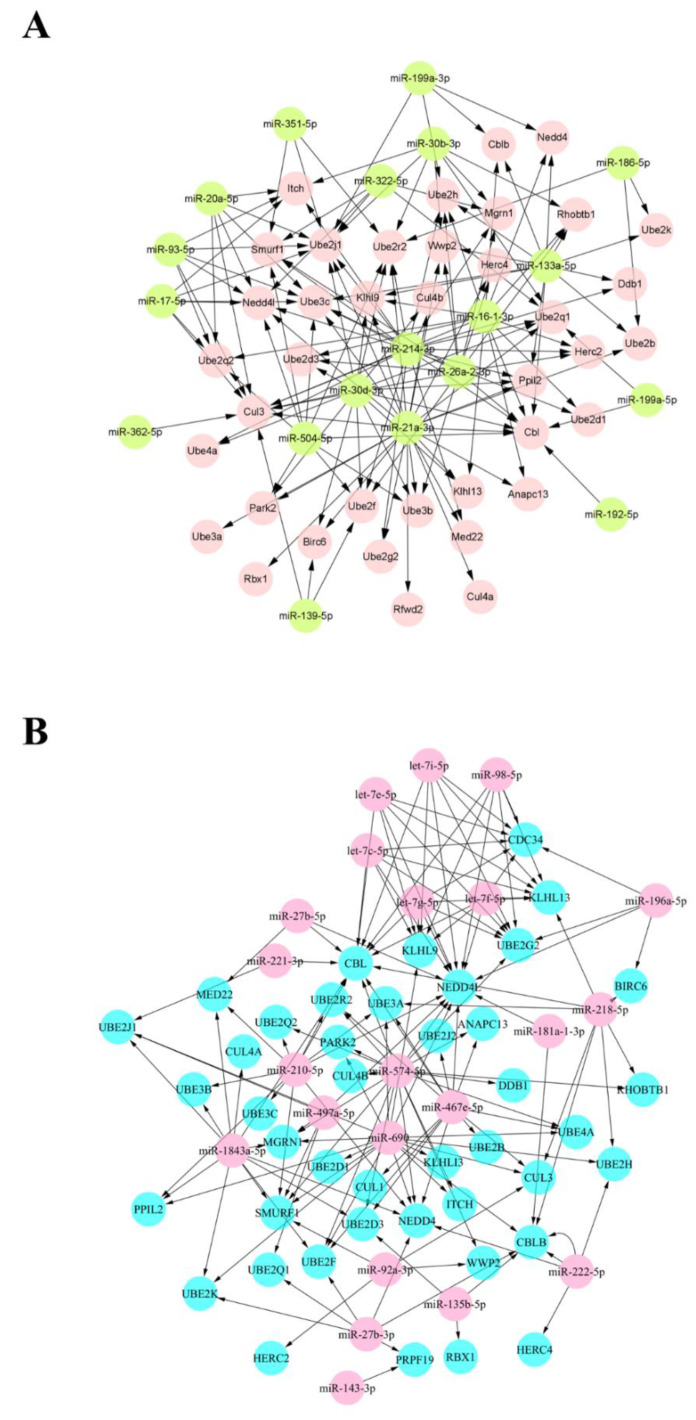
DEMs–DEGs interactive network. (**A**) Interaction network between upregulated DEMs and downregulated DEGs. (**B**) The interaction network between downregulated DEMs and upregulated DEGs.

In order to further reveal the miRNAs, genes, and the interaction between the two groups related to muscle atrophy, we conducted a joint analysis of DEMs and DEGs. It can be seen from the results of the analysis that 25 DEMs with upregulated expression can target 42 DEGs (Figure 3A), forming 151 target pairs. Similarly, 26 DEMs with downregulated expression can target 39 DEGs (Figure 3B), forming 152 target pairs. We constructed two miRNA-target gene interaction networks based on the target relationship between DEMs and DEGs obtained by data analysis. According to Figure 3, miR-27b-3p targets multiple DEGs and may play an important role. In addition, compared with other miRNAs, its abundance is higher. Therefore, it can be concluded that miR-27b-3p is a crucial node, with multiple common points as the target. Next, we took into consideration the candidate genes screened in mRNA sequencing. In the end, we speculate that miR-27b-3p/Cbl-b may be an essential regulator of muscle atrophy, and we will focus on the status of the two groups in muscle atrophy.

### 3.4. miR-27b-3p Can Affect Muscle Atrophy

**Figure 4:**
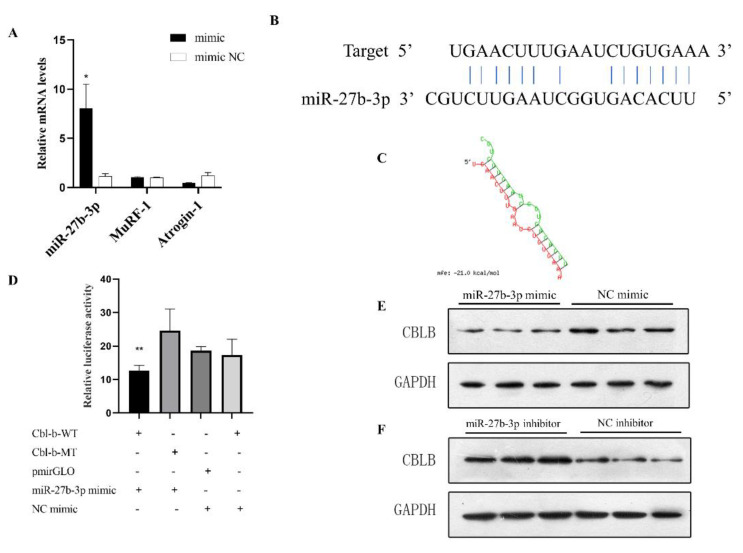
miRNA-27b-3p can affect muscle atrophy marker genes and target Cbl-b. (**A**) miR-27b-3p can relieve Dex-induced atrophy in myotubes. (**B**,**C**) Sequence of miR-27b-3p binding site with target gene. (**D**) The luciferase assay was performed by co-transfection of wild-type or mutant Cbl-b 3′UTR with miR-27b-3p mimic or mimic NC in C2C12 cells. (**E**,**F**) After transfection with miR-27b-3p mimic or inhibitor, Cbl-b protein expression was obtained. Results are shown as mean ± S.E.M. and the data are representative of at least three independent assays. Independent sample *t*-test was used to analyze the statistical differences between groups. (* *p* < 0.05; ** *p* < 0.01).

In order to explore the role of miR-27b-3p in the atrophic myotubes of C2C12, we first verified that the expression of miR-27b-3p is downregulated in the myotubes induced by the Dex treatment, suggesting that miR-27b-3p may play an active role in muscle atrophy. In order to further verify the effect of miR-27b-3p on muscle atrophy, we synthesized the miR-27b-3p mimic. The qPCR quantitative results showed that, compared with the control group, the miR-27b-3p mimic could significantly increase the abundance of miR-27b-3p in C2C12 cells. In particular, the treatment of Dex-induced atrophy of myotubes with the miR-27b-3p mimic can alleviate this induced atrophy to a certain extent (Figure 4A).

### 3.5. Cbl-b Is the Target Gene of miR-27b-3p

In the previous analysis and prediction, Cbl-b was the target gene of miR-27b-3p. We predicted the binding site of miRNA and target gene (Figure 4B,C), and performed a dual luciferase assay at the cellular level to verify the targeting relationship between miR-27b-3p and the 3′UTR of Cbl-b. The ratio of firefly luciferase/Renilla luciferase in the Cbl-b-WT and miR-27b-3p mimic group was significantly lower than that in the other three groups, indicating that miR-27b-3p could bind to the 3′UTR of Cbl-b (Figure 4D). Compared with other groups, the fluorescence intensity of the mimic and Cbl-b-WT plasmid co-transfection decreased, indicating that miR-27b-3p can indeed bind to Cbl-b 3′UTR. In addition, after the overexpression of miR-27b-3p, the protein level of Cbl-b showed a downward trend (Figure 4E). In the same way, the miR-27b-3p inhibitor binds to the endogenous miR-27b-3p and blocks its activity, and the protein expression level of Cbl-b is upregulated (Figure 4F). These results indicate that Cbl-b is the target gene of miR-27b-3p, and we speculate that miR-27b-3p plays a vital role in the regulation of muscle atrophy by influencing Cbl-b.

### 3.6. UpRegulation of Cbl-b Expression in Mice with Muscle Atrophy

**Figure 5:**
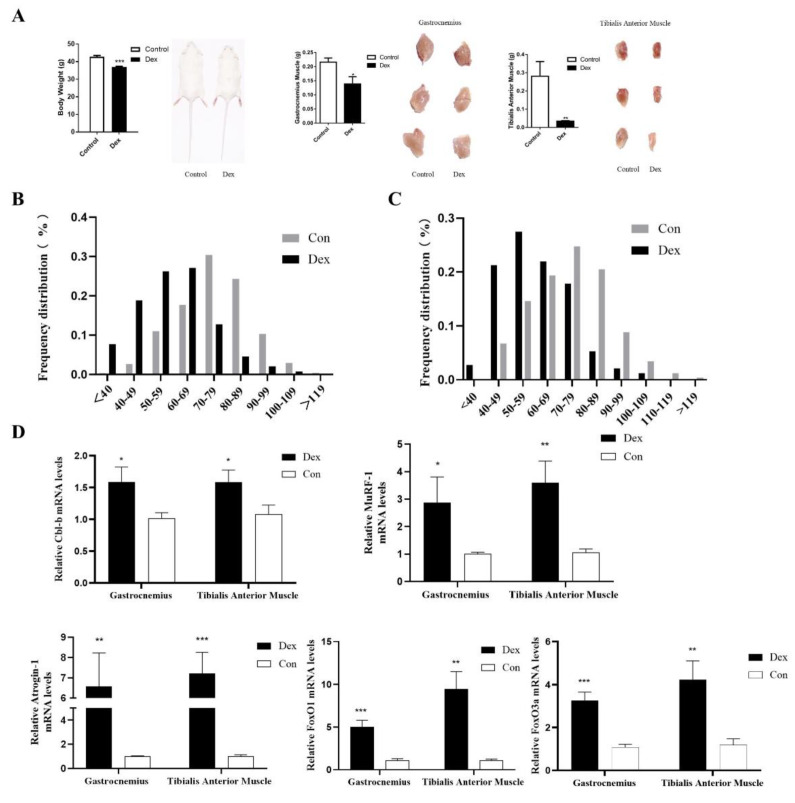
Some differences between the Dex group and Con group mice. (**A**) Differences in body weight, gastrocnemius weight, tibial anterior muscle weight, and morphology between the two groups of Kunming mice. (**B**) Diameter distribution of the gastrocnemius tube in the two groups. (**C**) Diameter distribution of the tibial anterior muscle tube in the two groups. (**D**) Differential expression of Cbl-b, Atrogin-1, Murf-1, FoxO1, and FoxO3 in the two groups of mice. Dex, dexamethasone. Con, control. Results are shown as mean ± S.E.M. and the data are representative of at least three independent assays. Independent sample *t*-test was used to analyze the statistical differences between groups. (* *p* < 0.05; ** *p* < 0.01; *** *p* < 0.001).

To verify the role of Cbl-b in muscle-wasting mice, we established a mouse model of muscle atrophy caused by dexamethasone treatment (Dex). Muscle atrophy was confirmed by increased body weight, gastrocnemius, and tibialis anterior muscle weights, myotube diameter, and associated atrophy genes, including Atrophy-1 and MuRF-1 in mice (Figure 5A–C). We found through qRT- PCR that Cbl-b expression was increased in mice with muscle atrophy (Figure 5D). In addition, the expression levels of FoxO1 and FoxO3 were also increased (Figure 5D). These results indicate that Cbl-b plays a role in muscle atrophy.

### 3.7. Cbl-b Gene Can Promote Muscle Atrophy

**Figure 6:**
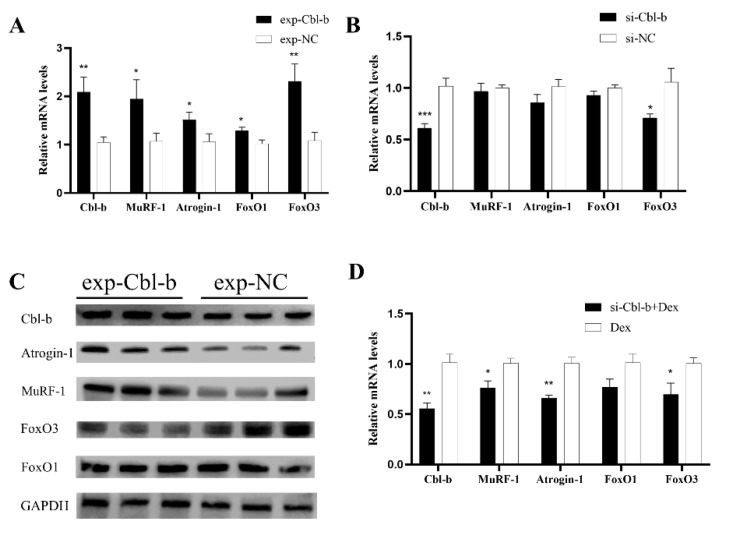
Cbl-b affects muscle atrophy. (**A**) Relative gene expression after the overexpression of Cbl-b. (**B**) Relative gene expression after losing function of Cbl-b. (**C**) Relative protein level after the overexpression of Cbl-b. (**D**) Relative gene expression after losing function of Cbl-b in induced muscle atrophy cells. Results are shown as mean ± S.E.M. and the data are representative of at least three independent assays. Independent sample *t*-test was used to analyze the statistical differences be-tween groups. (* *p* < 0.05; ** *p* < 0.01; *** *p* < 0.001).

To study the role of Cbl-b in muscle atrophy, we conducted gain and loss of function experiments of Cbl-b in C2C12 differentiated myotubes. After transfecting the Cbl-b overexpression plasmid into mouse C2C12 differentiated myotubes for 48 h, the overexpression of Cbl-b enhanced the mRNA expression levels of markers of myoblast atrophy, such as Atrogin-1, MuRF-1, FoxO1, and FoxO3 (Figure 6A). However, compared with overexpressing Cbl-b, there was no significant difference in the mRNA expression levels of Atrogin-1, MuRF-1, FoxO1, and FoxO3 after the transfection of Cbl-b siRNA in C2C12 cells (Figure 6B). Similarly, after the overexpression of Cbl-b, the protein expression levels of Atrogin-1, MuRF-1 and FoxO3 increased, but the protein expression levels of FoxO1 did not change (Figure 6C). The results further confirmed that Cbl-b plays a certain role in muscle atrophy.

### 3.8. Inhibition of Cbl-b Gene Expression Can Alleviate Muscle Atrophy

To explore whether the inhibition of Cbl-b gene expression can affect muscle atrophy, we used Dex to treat the myotubes 24 h after the transfection of Cbl-b siRNA in the C2C12-differentiated myotubes. Through RT-qPCR, it was found that, compared with the two groups that only used Dex treatment and blank control, in the treatment group added with siRNA, Cbl-b knockdown could significantly alleviate muscle atrophy (Figure 6D). The mRNA expression levels of relevant marker genes for muscle atrophy, such as Atrogin-1, MuRF-1, FoxO1, and FoxO3, were not significantly different compared with the blank control group.

## 4. Discussion

miRNAs are negative regulators of animal gene expression. miRNAs can bind to specific miRNA target sites in mRNA transcripts, usually located in animals’ 3′-UTR (untranslated region). When miRNAs bind to target sites on mRNA, they guide RNA-induced silencing complex (RISC) to these transcripts, silencing the messages carried by these mRNAs [22]. miRNA can inhibit the expression of target genes in two ways by organizing mRNA translation or causing it to be explained by RISC [22,23]. In previous studies, it was found that miRNAs are involved in various regulatory pathways in skeletal muscle [24,25,26,27,28,29,30]. For example, miR-696 has been confirmed to be a physical-activity-dependent miRNA. Compared with resting, its expression is significantly affected by physical activity. The reason is that it can participate in mouse skeletal muscle metabolism by regulating the expression of PGC-1α [31]. At the same time, there have been many analytical experiments on miRNA regulation on muscle atrophy. In mice with chronic kidney disease (CDK)-induced atrophy, the overexpression of miR-23a/miR-27a can reduce muscle loss and improve the grip strength of mice [32]. miR-29b can promote different types of muscle atrophy induced by denervation, Dex, fasting, aging, and cancer cachexia by targeting IGF-1 and PI3K (p85a) [5]. In this paper, we provide a new miRNA that affects muscle atrophy, namely miR-27b-3p.

In this study, we revealed the role of mmu-miR-27b-3p in the model of muscle atrophy induced by mouse myoblasts and found that it is based on regulating the expression of Cbl-b to affect muscle atrophy. The atrophy and maintenance of skeletal muscle is a process of protein decomposition and synthesis, and miRNA can regulate this process. In the existing research, the targeting of miR-27b to peroxisome proliferator-activated receptor gamma (PPARγ) is considered one of the mechanisms regulating lipid metabolism. It can regulate its expression by inducing adipose triglyceride lipase (ATGL) and lipoprotein lipase (LPL) post-transcriptional modification [33]. One of the main functions of PPARγ is to deposit fat in organs, especially for the deposition of fat in muscle tissue. There are two isoforms of PPARγ, PPARγ1 and PPARγ2 [34]. PPARγ1 is highly expressed in adipocytes and expressed to varying degrees in other tissues, such as the liver and colon. PPARγ2 is mainly expressed in adipose tissue and plays an important role in adipogenesis and triglyceride storage [35]. Skeletal muscle is a huge metabolic organ, and energy metabolism is an important part of its function. Studies have shown that rosiglitazone (RSG) reduces excessive lipolysis by reducing the expression of adipose triglyceride lipase (ATGL) and hormone-sensitive lipase (HSL) activity, thereby reducing free fatty acids release from insulin-resistant 3T3-L1 adipocytes. At the same time, after RSG treatment, insulin resistance in C2C12 myotubes co-cultured with insulin-resistant 3T3-L1 adipocytes was improved [36]. Moreover, studies have found that inhibiting lipolysis by knocking out adipose triglyceride lipase (ATGL) or hormone-sensitive lipase (HSL) can improve certain characteristics of cancer-related cachexia (CAC). Tumor-carrying ATGL-deficient mice can resist lipolysis of white adipose tissue, cardiomyocyte apoptosis, and increased proteasome muscle degradation, and maintain normal fat and gastrocnemius muscle mass [37]. In addition, during the process of disuse-induced muscle atrophy, the lipid accumulation and metabolism of specific fatty acids changes [38]. In summary, we speculate that PPARγ and ATGL may affect muscle atrophy through lipid metabolism. However, the direct link between PPARγ and ATGL and skeletal muscle atrophy remains unclear. Therefore, whether our research object miR-27b-3p can affect muscle atrophy through miR-27b-3p/PPARγ/ATGL pathway remains to be further studied. In chickens, miR-27b-3p significantly promoted the proliferation of chicken primary myoblasts (CPMs) by targeting myostatin (MSTN) and inhibited the differentiation of CPMs [39]. Interestingly, some studies have shown that in mice, excessive retinoic acid upregulates miR-27b-3p and targets α-dystrobrevin to inhibit myoblast proliferation and differentiation [40]. Furthermore, studies have found that miR-27b-3p is missing in breast cancer tissues and cell lines and plays a major role in sensitizing breast cancer cells to broad-spectrum anti-cancer drugs in vitro and vivo. miR-27b can enhance the response to paclitaxel by directly targeting Cbl-b and GRB2 to inactivate the PI3K/Akt and MAPK/Erk signaling pathways. In addition, miR-27b has been identified as a promising molecular biomarker in breast cancer patients’ chemoresistance, clinicopathological characteristics, and prognosis [41].

After changing the medium with 2% horse serum to induce differentiation for 4 days, myotubes appeared in C2C12 cells. After 24 h of treatment with dexamethasone, compared with normal myotubes, in the treatment group appeared atrophy that were not due to other reasons, such as lack of nutrients. Our research found that miR-27b-3p is downregulated in the model of muscle atrophy, which indicates that it has a potential role in the process of skeletal muscle atrophy. Through the combined analysis of DEMs and DEGs, miR-27b-3p co-targeted six genes in the DEGs results, namely UBE2K, UBE2Q1, UBE2F, NEDD4, Cbl-b, and PRPF19. These genes are some ubiquitin-conjugating enzymes, E3 ubiquitin-protein ligase, and RNA precursor processing factors, which are mainly involved in the modification and degradation of proteins and involve various biological functional processes. We found that the mature sequence of miR-27b-3p matches the 3′-UTR of Cbl-b. Through data analysis, we found that the 3′-UTR of Cbl-b can be stably combined with miR-27b-3p, and its seed-binding region remains conserved among multiple species. The results of dual-luciferase detection showed that miR-27b-3p has a direct targeting relationship with Cbl-b. After the overexpression or inhibition of miR-27b-3p, the expression level of Cbl-b also decreased and increased, respectively. It is worth noting that we did not directly transfect miR-27b-3p inhibitor in normal myotubes to verify whether it can cause muscle atrophy. Because we consider that the abundance of these small miRNA molecules is low, knocking down their expression level further may cause no significant changes in the results. At the same time, the Dual-luciferase Reporter Assay and Western blot analysis are highly feasible and accurate methods for detecting the relationship between miRNA and target gene, and they are widely used in related research in various fields.

It is well known that regulating FOXOs transcription factors through the PI3K/AKT pathway, thereby up-regulating the expression of the E3 ligase MuRF-1 and Atrogin-1, increasing the breakdown of muscle protein, and reducing the synthesis of muscle protein, is an important pathway in the process of muscle atrophy [42]. For example, it is reported that the IGF1/PI3K/Akt pathway can block the nuclear translocation of the FOXO transcription factor to block the upregulation of the atrophic ubiquitin connexin MuRF1 and Atrogin-1 induced by dexamethasone [43,44]. In this study, we confirmed in myotube function experiments that Cbl-b can affect the function of muscle atrophy. Cbl-b overexpression promotes the upregulation of FoxO1 and FoxO3a expression, which in turn promotes the upregulation of ubiquitin ligase MuRF1 and Atrogin-1 expression and promotes muscle atrophy. To further explore the effect of Cbl-b on muscle atrophy, we lost function of Cbl-b in the dexamethasone-induced atrophy model. The results show that losing function of Cbl-b can alleviate the muscle atrophy caused by dexamethasone.

To date, muscle atrophy is mainly intervened through physical exercise or nutritional support. Many studies show that exercise and nutritional support can affect muscle atrophy. For example, compared with sedentary older adults who do not exercise regularly, adults who exercise can significantly reduce their slow fiber diameter and improve their strength and mobility [45]. Older adults who exercise regularly have larger muscles and more slow fiber type groupings, proving that exercise can maintain slow motor neurons, thereby re-innervating muscle fibers [46]. In terms of nutritional support, protein supplementation can promote skeletal muscle protein synthesis under disused conditions and inhibit protein breakdown [47,48,49]. Although there are many promising therapeutic targets for treating muscle atrophy, it has been reported that the thyroid hormone can protect fasting-induced skeletal muscle atrophy by promoting metabolic adaptation [50]. However, no drug has been clinically proven to be safe. In summary, our research reveals a new way for mmu-miR-27b-3p to regulate Cbl-b and influence muscle atrophy. We believe that miR-27b-3p/Cbl-b may be a promising therapeutic target for muscle atrophy.

## 5. Conclusions

In summary, this study analyzed the differentially expressed miRNA and genes in muscle atrophy through miRNA sequencing and transcriptome sequencing and verified their target relationships through experiments. The results indicate that miR-27b-3p may play an important role in muscle atrophy, affecting muscle atrophy by regulating the Cbl-b gene. Our results lay the foundation for further research on the regulation mechanism of muscle atrophy and for the auxiliary treatment of muscle atrophy based on molecular drugs.

## Supplementary Materials

The following are available online at https://www.mdpi.com/article/10.3390/biom12020191/s1. Table S1: The mimic and inhibitor of miR-27b-3p and siRNAs used for RNA interference; Table S2: Primers used for qPCR; Table S3: Gene sequence of 3′UTR inserted in pmirGLO vector.
