# Supplementary material for: miR-27b-3p Attenuates Muscle Atrophy by Targeting Cbl-b in Skeletal Muscles
Source: Biomolecules. 2022 Jan 23;12(2):191. doi: 10.3390/biom12020191 (PMC8961554; doi:10.3390/biom12020191)
Supplement: Supplementary file 1 [file biomolecules-12-00191-s001.zip › biomolecules-1528373-supplementary.pdf]

Table S1. The mimic and inhibitor of miR-27b-3p and siRNAs used for RNA interference.

Fragment Name	Sequence (5′ to 3′)
mmu-miR-27b-3p mimic	UUCACAGUGGCUAAGUUCUGC
mmu-miR-27b-3p inhibitor	GCAGAACUUAGCCACUGUGAA
si-m-Cbl-b_001	CCACCGTATATACTTGAT
si-m-Cbl-b_002	GCAATATCCTACAGACCAT
si-m-Cbl-b_003	GCTCCGAGCAGGTTCTTAT
si-m-Cbl-b_004	ATCGAACATCCCAGATTTA
si-m-Cbl-b_005	GAGCATACT TCGAGAATTT
si-m-Cbl-b_006	TGAACCTACACCTCACGAT

Table S2. Primers used for qPCR.

Primer Name	Primer Sequences (5′ to 3′)	Size (bp)
Cbl-b	F: CGCATTTTGGGGATTATTG<br>R: GCTTGGGATTTTGGCACAG	133
Atrogin-1	F: GCAGAGAGTCGGCAAGTC<br>R: CAGGTCGGTGATCGTGAG	142
MuRF-1	F: AGTGTCCATGTCTGGAGGTCGTTT<br>R: ACTGGAGCACTCCTGCTTGTAGAT	139
FoxO1	F: TGGTGAAGAGCGTGCCCTACT<br>R: TTGCCCAGACTGGAGAGATGC	275
FoxO3	F: TCGTCTCTGAACTCCTTGCGT<br>R: TGGTGGAGCAAGTTCTGATTG	296
GAPDH	F: CACCATCTTCCAGGAGCGAG<br>R: CCTTCTCCATGGTGGTGAAGAC	101

Table S3. Gene sequence of 3′UTR inserted in pmirGLO vector

Name	sequences (5′ to 3′)
Wild-type	gagctcCCAGCTTCTCTGGTTCCACAGCTCTCTTAGGATGCCC<br>ACACTGAAGCTTCTGTGTTTGTGCTAGCCATACTTTGAAAT<br>CAGGGTTGAACTGATAAAATAATTTAAAGACGTTTACTCCC<br>CCTTGAACTTTGAATCTGTGAAATGCTTTTCCTTGTTTACAC<br>GTTGGCAGAATTGCAGTTTGTCTCTGTTTTTGATCCCTGTAC<br>TGTGTTCCTGACAGGCCCTTTGCAGAGTTGCTCAGGTCTGC<br>TGTAAGTTTCTCCATGCCTGCCCTGGTGctcgag
Mutated form	gagctcCCAGCTTCTCTGGTTCCACAGCTCTCTTAGGATGCCC<br>ACACTGAAGCTTCTGTGTTTGTGCTAGCCATACTTTGAAAT<br>CAGGGTTGAACTGATAAAATAATTTAAAGACGTTTACTCCC<br>CCTACGACTTCGAATCTGTGAAATGCTTTTCCTTGTTTACAC<br>GTTGGCAGAATTGCAGTTTGTCTCTGTTTTTGATCCCTGTAC<br>TGTGTTCCTGACAGGCCCTTTGCAGAGTTGCTCAGGTCTGC<br>TGTAAGTTTCTCCATGCCTGCCCTGGTGctcgag
